# Primiparity, class 3 obesity, intention to breastfeed and breastfeeding initiation

**DOI:** 10.1371/journal.pone.0322232

**Published:** 2025-04-29

**Authors:** Leandro Cordero, Michael R. Stenger, Bradley J. Needleman, Sabrena Noria, Mark B. Landon, Craig A. Nankervis

**Affiliations:** 1 Department of Pediatrics, College of Medicine, The Ohio State University Wexner Medical Center, Columbus, Ohio, United States of America; 2 Department of Surgery, College of Medicine, The Ohio State University Wexner Medical Center, Columbus, Ohio, United States of America; 3 Department of Obstetrics and Gynecology, College of Medicine, The Ohio State University Wexner Medical Center, Columbus, Ohio, United States of America; University of Cambridge, UNITED KINGDOM OF GREAT BRITAIN AND NORTHERN IRELAND

## Abstract

**Background:**

During the 2013–21 period, 674 primiparous women with Class 3 obesity delivered in our institution. Their antenatal infant feeding preference on admission was: 518 (77%) intended to breastfeed (BF) only, 101 (15%) intended to feed formula only and 55 (8%) intended to BF and formula feed combined. Intention to BF only is a predictor of BF initiation, however, data concerning this relationship is limited.

**Objective:**

To determine the perinatal variables that influence success or failure of BF among primiparous women with Class 3 obesity who antenatally declared their intention to only BF.

**Methods:**

Retrospective cohort study of women who delivered live singletons without major malformations at ≥ 34 weeks gestation.

**Results:**

The 518 women who prenatally intended to BF only, were categorized at discharge as exclusive BF *(EBF)* 197 (38%), any BF (*ABF*) 212 (41%) and formula feeding (*FF*) 109 (21%). A lower prevalence of gestational diabetes (10,17 vs 17%), chronic hypertension (16,31 vs 18%), severe preeclampsia (12,22 vs 30%), cesarean delivery (37,55 vs 55%), late preterm (6,17 vs 14%), neonatal hypoglycemia (8,26 vs 22%) and NICU admission (11,25 vs 20%) was present in the *EBF* group as compared to *ABF* and *FF*. African American women were more likely to only *FF*. Regression analysis showed that the stronger predictors of failure to initiate BF were public healthcare assistance, African American race, preeclampsia, cesarean delivery, neonatal hypoglycemia, prematurity and NICU admission.

**Conclusion:**

Among primiparous women with class 3 obesity who intended to BF only but failed to do so, the increased prevalence and severity of maternal and neonatal morbidities are likely obstacles to BF their first infant.

## Background

Obesity is the most common medical condition that affects women of reproductive age across the world [1–3]. The prevalence of obesity among women 20–39 years of age increased from 29.8% in 2001–2002 to 39.7% in 2017–2018 [1]. The benefits of lactation on short and long term maternal and infant health have been clearly documented [4–6]. Specifically, exclusive breastfeeding *(EBF)* or any breastfeeding *(ABF)* during birth hospitalization and through the first postpartum year are important for both healthy women as well as for those with comorbidities [6–10]. Few studies on perinatal outcomes including BF for women with Class 3 obesity (pregestational body mass index (BMI) 40–49 kg/m^2^ *morbid* and ≥ 50 kg/m^2^ *extreme*) have been reported [11–13]. Recently, an inverse relationship between BMI and *ABF* has been established [14]. Intention to BF only is a strong predictor, although not absolute, of BF success, however, there is limited information on the proportional rates of *EBF, ABF* or formula feeding (*FF*) at discharge among primiparous women with Class 3 obesity [10,13].

A positive BF experience for a woman, primiparous or multiparous, will improve her chances for success in the next pregnancy, however an unsuccessful experience will lower the probability of BF subsequent infants [15]. Considering the above, it is important to identify the obstacles that lead to BF failure in primiparous women with Class 3 obesity who intended to BF only.

## Objective

To determine perinatal variables that may influence the achievement of *EBF* and *ABF* among 518 primiparous women with Class 3 obesity who declared prenatally their intention to BF only.

## Subjects and methods

This retrospective study of medical records was approved by the Biomedical Sciences Institutional Review Board of The Ohio State University (IRB 2010H0198) with waivers of informed consent and HIPAA research authorization, in accordance with the relevant guidelines and regulations of the declaration of Helsinki. The de-identified and coded data from electronic maternal and neonatal records (2013–2021) were reviewed. According to their pregestational BMI, women were classified as normal (BMI 18.5–24.9 kg/m^2^), overweight (BMI 25–29.9 kg/m^2^), obese Class 1 (BMI 30–34.9 kg/m^2^), Class 2 (BMI 35–39.9 kg/m^2^) or Class 3 (divided into *morbid* obesity BMI 40–49 and *extreme* obesity ≥ 50 kg/m^2^) [2,14]. The study population was composed of women with Class 3 obesity who delivered at ≥ 34 weeks of gestation singleton live births without major malformations. Infants born between 34 and 36 6/7 weeks were considered late prematures.

During their prenatal care visits, on arrival to Labor and Delivery and shortly after delivery, women were asked about their anticipated infant feeding preference (BF only, BF and FF combined or *FF* only) [10,13]. Gestational diabetes mellitus (GDM), Type 1 and Type 2 DM, chronic hypertension (CHTN), severe preeclampsia, anemia, polycystic ovary syndrome (PCOS), obstructive sleep apnea (OSA) and gastroesophageal reflux disease (GERD) were diagnosed and treated following established guidelines [16–18]. Gestational weight gain (GWG) was categorized as adequate, inadequate or excessive [19]. Clinical and demographic data from some women included in this investigation have been reported earlier [10,13,20].

Women were also informed of our hospital practices that encourage BF including early BF and formula supplementation only if medically indicated, rooming in and availability of full-time lactation consultants and post discharge BF support [10,13,20,21]. As required for hospital accreditation, our institution reports BF data to the Joint Commission [7].

Following delivery, symptomatic infants were transferred directly to the neonatal intensive care unit (NICU) for further care. Holding, skin-to-skin contact, and BF were encouraged depending on the mother-infant dyad condition. Asymptomatic infants able to feed were taken to the Newborn Nursery for routine care and glucose monitoring if indicated [10,13,20,21]. Using intrauterine growth charts, infants were identified as appropriate for gestational age (AGA), small (SGA), large (LGA) or macrosomic (birthweight ≥ 4000g) [10,13,20,21].

If indicated, blood glucose was assessed with Accu-Chek^®^ or by plasma glucose measurements starting during the first hour of life. Neonatal hypoglycemia was defined by blood glucose of < 40 mg/dl before 4 hours of life and by < 45 mg/dl thereafter. Infants with hypoglycemia were treated with glucose gel, BF or formula feeding and those with persistent hypoglycemia were transferred to the NICU for further care [10,13,20,21]. *EBF* was defined as direct feedings from the breast, expressed breast milk or donor human milk (DHM) [10,13,20,21]. *ABF* consisted of direct BF, expressed breast milk or DHM supplemented with formula [10,13,20,21]. BF was considered initiated if, during the 24 hours preceding hospital discharge, infants were *EBF* or *ABF* [10,13,20,21]. Due to the study design, information on post-discharge infant feeding was not available.

## Statistical analysis

Comparisons of BF outcomes (*EBF*, *ABF* or *FF*) were made with T-Tests for continuous variables following a normal distribution, and Wilcoxon Two-Sample tests for continuous variables that were not normally distributed. Chi-square analysis was used for categorical variables. Multivariate logistic regressions were used to determine the probability of BF initiation and the probability of *EBF* at discharge. Explanatory variables in both logistic models included BMI, DM, CHTN, race, healthcare public assistance, mode of delivery, infant feeding intention, fetal growth, preeclampsia, late prematurity, macrosomia, hypoglycemia, and admission to the NICU. Logistic models used backward elimination method for predictor selection, although forward and stepwise selection methods produced the same results. Significance was established at a *p*-value < 0.05. Analyses were performed with SAS version 9.4 (Cary, NC).

## Results

### Perinatal outcomes of women who intended to BF only

The study population was composed of 518 primiparous women with Class 3 obesity who prenatally declared their intention to BF only. Based on infant feeding at discharge, the 518 mother-infant dyads were divided into *EBF* (38%), *ABF* (41%) and *FF* (21%) (Table 1). The three groups were similar in age at delivery and current and former smoking combined but were different in rate of advanced maternal age, white race and public healthcare assistance.

**Table 1:** Perinatal outcomes of women who intended to breastfeed only.

Infant Feeding at Hospital Discharge	Exclusive Breastfeeding	Any Breastfeeding	Formula Feeding	*p*-value
Mother-Infant Dyads no. (%)	197 (38)	212 (41)	109 (21)	<0.0001^^^
BMI 40–49 kg/m^2^ no. (%) (Morbid)	113 (37)	105 (50)	50 (46)	0.1102^^^
BMI ≥ 50 kg/m^2^ no. (%) (Extreme)	84 (43)	107 (50)	59 (54)	
Mothers age (y) median[IQR]	28 [25,31]	28 [25,32]	27 [23,32]	0.3720^*^
Advanced maternal age no. (%)	23 (12)	27 (13)	21 (19)	0.1569^^^
Race: White no. (%)	155 (79)	153 (72)	53 (48)	**<0.0001** ^†^
African American no. (%)	37 (19)	48 (23)	51 (47)	
Hispanic no. (%)	3 (2)	4 (2)	2 (2)	
Other no. (%)	2 (1)	7 (3)	3 (3)	
Public Healthcare assistance no. (%)	65 (33)	88 (42)	69 (35)	**<0.0001** ^^^
Smoking: Current no. (%)	4 (2)	11 (5)	8 (7)	0.0941^^^
Former no. (%)	41 (21)	45 (21)	30 (15)	
Gestational Diabetes no. (%)	19 (10)	37 (17)	18 (17)	0.0529^^^
A1 no. (%)	6 (3)	17 (8)	4 (4)	
A2 on medication no. (%)	13 (7)	20 (9)	14 (13)	
Pregestational diabetes no. (%)	7 (4)	20 (10)	14 (13)	**0.0260** ^†^
Type 1 no. (%)	2 (1)	4 (2)	3 (3)	
Type 2 no. (%)	5 (3)	16 (8)	11 (10)	
Hypertensive disorders no. (%)	66 (34)	111 (52)	59 (54)	**0.0366** ^^^
Gestational hypertension no. (%)	35 (18)	44 (21)	24 (22)	
Chronic hypertension no. (%)	18 (9)	43 (20)	21 (11)	
Chronic hypertension meds no. (%)	13 (7)	24 (11)	14 (7)	
Preeclampsia severe features no. (%)	24 (12)	47 (22)	33 (30)	**0.0005** ^^^
Polycystic ovarian syndrome no. (%)	15 (8)	17 (8)	8 (4)	0.9744^^^
Obstructive sleep apnea no. (%)	6 (3)	9 (4)	9 (5)	0.1089^^^
Gastroesophageal reflux no. (%)	19 (10)	11 (5)	12 (6)	0.1176^^^
Asthma no. (%)	33 (17)	34 (16)	25 (13)	0.2771^^^
Anemia no. (%)	35 (18)	26 (12)	20 (11)	0.2109^^^
Postpartum hemorrhage no. (%)	9 (5)	4 (2)	4 (4)	0.3044^^^
Delivery: Primary cesarean no. (%)	72 (37)	117 (55)	60 (55)	**0.0002** ^^^
Vaginal no. (%)	125 (63)	95 (45)	49 (45)	
Pregestational weight kg median[IQR]	116 [99,130]	115 [99,136]	122 [99,136]	0.5913^*^
Weight at delivery kg median[IQR]	128 [116,144]	131 [116,148]	136 [118,155]	0.1047^*^
Weight gain kg median [IQR]	14 [10,20]	15 [9,23]	18 [10,25]	0.1357^*^
Excessive no. (%)	156 (79)	168 (79)	87 (80)	0.9994^^^
Adequate no. (%)	32 (16)	35 (17)	17 (16)	
Inadequate no. (%)	9 (5)	9 (4)	5 (4)	
Weight loss kg median[IQR]	-4 [-6,-3]	-3 [6,-2]	-4 [-4,-2]	0.6917^*^
Mother hospital stay (d) median[IQR]	4 [3,4]	4 [3,5]	4 [3,5]	**0.0028** ^*^

Analysis: ^†^ Fisher’s Exact Test, ^^^ Chi-Square, ^*^ Kruskal-Wallis Test

The rate of GDM (A1 combined with A2) was lower in the *EBF* group than in the other 2 groups (10,17 vs 17%) and Type 2 DM was also lower (3,8 vs 10%). CHTN with and without medication combined was higher in the *ABF* (31%) than in the other two groups (16 vs 18%). Severe preeclampsia was less prevalent in the *EBF* group (12,22 vs 30%), however, it was superimposed on diabetes mellitus in 25% of the *EBF*, 43% of the *ABF* and 42% of the *FF* group. Concurrently, preeclampsia was also superimposed on CHTN in 33% of *EBF*, 53% of *ABF* and 45% of *FF* group. Equally prevalent were OSA (3,4 vs 5%), PCOS (8,8 vs 4%), GERD (10,5 vs 6%), asthma (17,16 vs 13%) and anemia (18,12 vs 11%).

Vaginal delivery was more frequent in the *EBF* group (63,45 vs 45%) while primary cesarean was more common in the *ABF* and *FF* groups (37,55 vs 55%). Median pregestational weight (116,115 vs 122 kg), median weight at delivery (128,131 vs 136 kg) and rate of excessive (79,79 vs 80%), adequate (16,17 vs 16%) and inadequate GWG (5,4 vs 4%) were similar in all groups.

### Neonatal outcomes in the exclusive, any breastfeeding and formula feeding groups

Infants born to the 197 women from the *EBF,* 212 infants from the *ABF* and 109 from the *FF* group were comparable in male gender (45,58 vs 51%), median birth weight (3500,3394 vs 3326g), AGA (77,74 vs 76%), LGA (19,22 vs 20%) and SGA (4,4 vs 4%), but differed in median GA (39,38 vs 38w), late prematurity (6,17 vs 14%), hypoglycemia (8,26 vs 22%), admission to the NICU (11,25 vs 20%), and infant length of stay (2,3 vs 3d) (Table 2).

**Table 2:** Neonatal outcomes in the exclusive, any breastfeeding and formula feeding groups.

	Exclusive Breastfeeding	Any Breastfeeding	Formula Feeding	*p*-value
Mother-Infant Dyads no. (%)	197 (38)	212 (41)	109 (21)	0.0001^^^
BMI kg/m^2^ median[IQR]	46.5 [42,52]	50.0 [43,53]	50.4 [43,56]	**0.0080** ^*^
Gender (male) no. (%)	89 (45)	123 (58)	56 (51)	**0.0342** ^^^
Gestational age (w) median[IQR]	39 [38,40]	38 [37,39]	38 [37,39]	**<0.0001** ^*^
Late preterm (34–36wks) no. (%)	11 (6)	35 (17)	15 (14)	
Birthweight (g) median[IQR]	3500 [3219,3779]	3394 [2918,3739]	3326 [3020,3779]	0.0700^*^
Intrauterine Fetal Growth				
Appropriate for gestation no. (%)	151 (77)	156 (74)	83 (76)	0.9361^^^
Small for gestation no. (%)	7 (4)	9 (4)	4 (4)	
Large for gestation no. (%)	37 (19)	47 (22)	22 (20)	
Macrosomia no. (%)	25 (13)	30 (14)	16 (15)	0.8631^^^
Neonatal hypoglycemia no. (%)	15 (8)	56 (26)	24 (22)	**<0.0001** ^^^
Admission to NICU no. (%)	21 (11)	53 (25)	22 (20)	**0.0008** ^^^
Infant length of stay (d) median[IQR]	2 [2,3]	3 [2,4]	3 [2,3]	**<0.0001** ^*^
Lactation Assistance no. (%)	197 (100)	204 (96)	86 (79)	**<0.0001** ^^^
Time to the first breastfeeding				
1-2 hours no. (%)	130 (66)	78 (37)	10 (9)	**<0.0001** ^^^
3-6 hours no. (%)	51 (26)	64 (30)	17 (16)	
7-24 hours no. (%)	10 (5)	30 (14)	13 (12)	
≥ 25 hours no. (%)	6 (3)	40 (19)	14 (13)	
Never breastfed no. (%)	0 (0)	0 (0)	55 (50)	
Breastfeeding Initiation no. (%)	197 (100)	212 (100)	0 (0)	**<0.0001** ^^^

Analysis: ^^^ Chi-Square, ^*^ Kruskal-Wallis Test

Of the 96 infants admitted to the NICU, two thirds were transported directly from the delivery room while the other third remained with their mothers briefly before transfer. Considering the similarities between the three feeding groups, the main NICU admission diagnoses combined were respiratory distress (42%), hypoglycemia (34%), prematurity (19%) and miscellaneous (5%).

### Exclusive, partial and formula feeding at discharge

Of the 518 women who intended BF only, 197 (38%) *EBF,* 212 (41%) *ABF* and 109 (21%) *FF* at the time of hospital discharge (Table 2). There were 268 women with *morbid* (BMI 40–49 kg/m^2^) and 250 others with *extreme* obesity (BMI ≥ 50 kg/m^2^). The latter was lower in *EBF* (33 vs 40%) and higher in *FF* (24 vs 18%), however, these differences were not statistically significant. Lactation support was accepted by every woman in the *EBF*, by 96% of those in the *ABF* and by only 79% of those in the *FF* group. DHM contributed to BF in 22% of the *EBF* and to 8% of the dyads in the *ABF* group.

BF during the first 2 hours of life occurred in 66% of infants in the *EBF*, 37% in the *ABF* and only in 9% of infants in the *FF* group. In contrast, among women with severe preeclampsia, BF in the first two hours was recorded in 46% of *EBF*, 21% for those in *ABF* and 6% of the *FF* group. First BF occurred between 3 and 6 hours among 26% of women in the *EBF*, 30% on the *ABF* and 16% of the *FF* group. The remaining women in the *EBF*, *ABF* and one half of those in the *FF* group BF within 48 hours after birth. Unfortunately, the other half of those in the *FF* group never BF (Table 2). Eleven of the 197 (6%) infants in the *EBF* group had a formula supplement prescribed during the first 6 hours and 20 (10%) other infants during the first 24 hours. Concurrently, formula supplementation was prescribed within the first 6 hours in 57% of the infants in the *ABF* and in 77% of those who *FF* at discharge. In the *ABF* group on the day of discharge 31 (15%) of the 212 mothers BF three times while 104 (49%) others BF four times or more.

Regression analysis confirmed that race other than white (aOR 0.483 CI 95% 0.301, 0.775), public healthcare assistance (aOR 0.507 CI 95% 0.329, 0.780), and neonatal hypoglycemia (aOR 0.361 CI 95% 0.186, 0.703) were associated with *EBF* failure (Table 3). Concurrently late prematurity and admission to the NICU were significant obstacles to *EBF* (Table 2). In contrast, the likelihood of *EBF* increases with increasing weeks of gestational age (aOR 1.241 CI 95%1.023, 1.506) (Table 3). Regression analysis showed that African American women (aOR 0.327 CI 95% 0.198, 0.541), those who received public healthcare assistance (aOR 0.406 CI 95% 0.248, 0.663) or who had severe preeclampsia (aOR 0.486 CI 95% 0.265, 0.894) were less likely to initiate BF at discharge (Table 3).

**Table 3:** Logistic regressions for exclusive and any breastfeeding.

Exclusive Breastfeeding				
Predictors	*a Odds Ratio*	*95% LCL*	*95% UCL*	*p-*value
African American	0.483	0.301	0.775	0.0026
Public Healthcare Assistance	0.507	0.329	0.780	0.0020
Cesarean delivery	0.550	0.361	0.840	0.0056
Neonatal hypoglycemia	0.361	0.186	0.703	0.0027
Gestational age	1.241	1.023	1.506	0.0289
Any Breastfeeding				
Predictors	*a Odds Ratio*	*95% LCL*	*95% UCL*	*p-*value
African American	0.327	0.198	0.541	<0.0001
Healthcare Public Assistance	0.406	0.248	0.663	0.0003
Preeclampsia severe	0.486	0.265	0.894	0.0203

Neither iron deficiency anemia, postpartum hemorrhage nor intraoperative bleeding were independent predictors of BF success. Similarly, regardless of infant feeding outcomes, excessive, adequate or inadequate rates of GWG, were not independent predictors of *EBF* or *ABF* in this investigation.

## Discussion

Maternal preference for feeding their infants after birth included BF only, *FF* only or BF and *FF* combined (*ABF*). Although intention to BF is the preferable choice, it is not always a predictor of BF initiation [10,22–24]. In the past we have consistently reported discordance between intention to BF and achievement of BF outcomes [10,25–29]. The current analysis of 518 primiparous women with Class 3 obesity who intended BF only, showed that the rates of 38% *EBF*, 41% *ABF* and 21% *FF* at discharge are consistent with those we have reported in other high risk obstetrical populations [10,13,20,28,29].

The American Academy of Pediatrics, The Academy of Breastfeeding Medicine and the World Health Organization recommend *EBF* for all infants during birth hospitalization and beyond [7–8]. As recently as 2019, *EBF* was reported in the U.S. to be 62.6% at the time of discharge [9]. These rates are indeed quite different from those of 47% for infants born to women with CHTN and severe obesity [27], 37% with severe preeclampsia [13] and 19% with CHTN superimposed on PGDM [28]. Considering the above, the 38% *EBF* rate reported here for women who intended to BF only although disappointing is not surprising. Conrey et al. suggested that to improve *EBF* and BF duration one must focus on strengthening BF intention and identify effective interventions to support women with comorbidities and higher pregestational BMI [24].

Antenatal intention to provide BF and *FF* instead of BF only, at least in our experience, leads to lower rates of *EBF* and higher rates of *ABF* and *FF* at hospital discharge [20,29]. The results of the current study highlight the discordance between intention to BF only and *EBF* and BF initiation among women with high-risk obstetrical conditions including obesity [27–29]. The experience of 212 women who intended to BF only, but provided *ABF* at discharge instead could offer, however, a somewhat optimistic perspective since these women were able to BF 66% of their babies within 6 hours from birth and at the time of discharge, 135 (64%) of the infants were still BF up to four times per day. It is also possible that some of these women with support and encouragement could reinitiate and maintain *EBF* longer [10,20,24].

According to standard definitions, *EBF* and *ABF* infants combined represent a BF initiation rate of 79% for the 518 primiparous women with Class 3 obesity who prenatally intended to BF only [7–9]. Furthermore, in subsequent pregnancies these women will acknowledge a prior BF experience. The importance of this information is supported by multiple studies that showed that prior BF experience is a very strong predictor of BF initiation [15,30,31]. In a recent study of multiparous with PGDM we reported intention to BF only in 79% of 170 women with prior experience and in 46% of 134 others without prior BF experience [31]. Concurrently, their BF initiation following the subsequent pregnancy was 81% in the prior BF group and only 36% among those without prior experience [31].

While abundant literature addresses the impact of intention to BF on BF initiation, the reasons why women who intended to BF only succeed or fail are unclear. *FF* at discharge, by 109 (21%) of women in the current study is concerning because none of them changed their decision even after interactions with lactation consultants [10,13,20,29–31].

Time to the first BF is important because many critical maternal and neonatal physiological interactions occur during the first hour after birth [28,32–34]. In a recent study of infants born to women with PGDM, we reported that rates of *EBF* and of BF initiation at discharge were similar for infants who BF at birth or during the first six hours while those who BF later had lower rates of either type of BF [10]. The data presented here showed that the time of the first BF strongly associates with *EBF* at discharge as compared to those infants in the *ABF* and *FF* groups. Conversely, *FF* shortly after birth or during the hospitalization remain a strong predictor of BF initiation failure [35]. While early BF is preferable, maternal complications, neonatal morbidities or maternal-infant separation often preclude or delays BF initiation [21,32,33]. Formula supplementation even when medically indicated is an obstacle to *EBF* and BF initiation [32–35]. However, parents and health providers should be reminded that temporary formula supplementation needs not to deter resumption or continuation of BF [32–35].

Hypertensive disorders of pregnancy are common complications especially among women with severe obesity [13,26–29]. The data presented here showed a strong association between severe preeclampsia and failure to establish *EBF* (18%) or *ABF* (54%) (a OR 0.486 CI 95% 0.265, 0.894). The adverse effects of preeclampsia are also compounded by its severity, by the superimposition of CHTN and DM, by the need for magnesium sulfate treatment or by the often urgency for cesarean delivery [13,18,36,37].

Our data also showed that cesarean delivery was a strong predictor of *EBF* failure (a OR 0.550 CI 95% 0.361, 0.840). Delays in BF initiation accompanying cesarean delivery are associated with maternal-infant separation, reduced sucking ability and insufficient milk supply, all factors predictive of low BF initiation and shortened BF duration [36–39]. Furthermore, some of these obstacles may be compounded by delayed lactogenesis, especially in women with severe obesity [38]. Regardless of the mechanism, mother-infant separation undoubtedly has an emotional impact on the mother and a likely physiological effect on mother and infant [37–39].

The rate of NICU admission among the *ABF* and *FF* groups was higher than in the *EBF* group. NICU admission has been a global marker of undesirable perinatal outcomes and low BF initiation and duration for women with complex pregnancies such as DM, CHTN, preeclampsia and obesity [13,21,28,29,31]. As reported in the current manuscript, the number of NICU admissions due to hypoglycemia is lower than that reported in the past [21,32,33]. The treatment of hypoglycemia by early BF, *FF* or glucose gel administration in the Newborn Nursery may have prevented the need for intravenous glucose infusions given at the NICU [21,32,33]. Changes in our neonatal practices decreased the number of asymptomatic infants born to women with PGDM admitted to the NICU from the 2008–11 to the 2013–16 period, concurrently, *EBF* increased from 13 to 24% and *ABF* from 45 to 60%, respectively [33]. In spite of the progress made, the prevalence of late prematurity and the unavoidable mother-infant separation created by the NICU admission remained significant obstacles to BF [21,33].

Our data shows that socio-economic and racial discrepancies are associated with low rates of *EBF* and BF initiation in African Americans as compared to non-Hispanic Whites. In light of these findings, we agree that although BF rates in the U.S. have improved in the last decade, racial/ethnic disparities persist [40]. Additionally, we also acknowledge that maternal and child health is significantly influenced by a variety of factors including but not limited to race, ethnicity, education, insurance and multiple comorbidities, and that major efforts are still needed to improve BF and BF duration rates in the U.S [25-26, 40-41].

This investigation is limited by the lack of an infant feeding intention scale and follow-up information regarding infant feeding after discharge from the hospital. Also, the definition of BF initiation at discharge may be only applicable to women with complex pregnancies or whom early mother-infant contact may be delayed. The strength of this investigation rests on the size of the high risk obstetrical and neonatal population and the fact that the data were obtained directly from hospital records not via post-delivery maternal questionnaires.

## Conclusion

In summary, we provided further evidence that intention to BF only is not an absolute predictor of *EBF* and of BF initiation for pregnancies complicated by severe obesity alone or superimposed on CHTN, preeclampsia and diabetes mellitus. Late prematurity, admission to NICU, neonatal hypoglycemia and racial and economic disparities remain obstacles to BF initiation. Although the rate of *EBF* and *ABF* reported here may seem disappointing, they still provide some optimism because multiparous women with prior BF experience are more likely to provide *EBF* or *ABF* in subsequent pregnancies. The fact that approximately twenty percent of primiparous women who intended to BF only failed to do so and *FF* their infants at hospital discharge represents a major challenge. Recognition that among women who intend to BF only, especially those in high-risk populations, a significant number fail to achieve their goal should prompt the implementation of strategies prior to discharge from the hospital to further successful BF initiation.

## Supporting information

S1 Data Raw data. (XLSX)
